# Vibrio fluvialis cholangitis with bacteremia and refractory septic shock: a case report and review of the literature

**DOI:** 10.1007/s15010-025-02535-7

**Published:** 2025-05-16

**Authors:** Roberto Lazzari, María Borja Cano, Alba Rivera Martínez, Marc Rubio Bueno, Marta Castella Rovira, Mireia Puig Campmany

**Affiliations:** 1 Emergency Department, Hospital de la Santa Creu I Sant Pau, Barcelona, Spain; 2 Microbiology Department, Hospital de la Santa Creu i Sant Pau, Barcelona, Spain; 3 Sant Pau Institute of Biomedical Research (IIb Sant Pau), Barcelona, Spain; 4 Genetics and Microbiology Department, Universitat Autònoma de Barcelona, Barcelona, Spain; 5 Department of Medicine, Universitat Autònoma de Barcelona, Barcelona, Spain

**Keywords:** Bacteremia, Septic shock, Vibrio, Cholangitis

## Abstract

*Vibrio fluvialis* is an emerging pathogen primarily associated with gastroenteritis, though an increasing number of extraintestinal infections have been reported. We present the first documented case in Europe of *V. fluvialis* cholangitis with liver abscess and bacteremia. An 85-year-old man with diabetes mellitus and chronic steroid use was admitted with severe epigastric pain but no fever or gastrointestinal symptoms. Initial laboratory tests were unremarkable, yet imaging revealed a hepatic abscess. Despite early antibiotic therapy, the patient rapidly developed refractory septic shock and died within 12 h. Blood cultures confirmed *V. fluvialis*. This case highlights the potential for severe *V. fluvialis* infections even in the absence of known seafood or seawater exposure. Given the global rise in raw seafood consumption, physicians should consider *V. fluvialis* as a potential pathogen in diabetic or immunocompromised patients presenting with hepatobiliary infections and sepsis.

## Introduction

*Vibrio* species are Gram-negative, straight or curved rod-shaped, motile, and facultative anaerobic bacteria that are natural inhabitants of aquatic ecosystems including freshwater, estuarine, and marine environments [1]. Several species are known to cause infections in humans, of which *Vibrio cholerae*, *Vibrio parahaemolyticus*, *Vibrio vulnificus*, and *Vibrio alginolyticus* are considered the most significant [2].

*Vibrio* spp. infections have traditionally been classified into cholera and non-cholera. Cholera is an acute diarrheal disease primarily caused by the consumption of water or food contaminated with toxigenic *V. cholerae* (serogroups O1 and O139). While it is considered a rare disease in developed countries, it remains endemic in regions of Asia and Africa, a situation exacerbated by the improper quality of drinking water [3]. Non-cholera *Vibrio* spp. infections typically arise from the consumption of raw fish and undercooked seafood or from direct exposure to contaminated water. These non-cholera infections are primarily characterized by gastroenteritis and wound infections, and can progress to systemic infections, particularly among patients with predisposing health conditions such as hepatic disease, diabetes mellitus, and immunosuppression [2].

*Vibrio fluvialis* has been described as an emerging pathogen in diverse regions across the globe [4, 5]. According to a recent systematic review and meta-analysis, *V. fluvialis* was the third most prevalent among non-cholera *Vibrio* species in South Asia, behind non-O1/non-O139 *V. cholerae* and *V. parahaemolyticus* [6]. The U.S. Centers for Disease Control and Prevention reported *V. fluvialis* as the fifth most prevalent in 2019, with 110 cases registered [7]. In Europe, *V. fluvialis* is uncommon, and sporadic cases of gastroenteritis or wound infections have been described [8–10].

We report a fatal case of *V. fluvialis* cholangitis with liver abscess and bacteremia in a patient without any evident history of consuming raw or undercooked fish or seafood, or exposure to brackish aquatic environments.

## Case report

An 85yo man with medical history of hypertension, diabetes mellitus chronic obstructive pulmonary disease, pulmonary fibrosis, atrial fibrillation, dementia punctuated as 5 in the Global Deterioration Scale and polypharmacy which included chronic steroids was brought to the Emergency Department (ED) by the pre-hospital emergency service for severe epigastric pain of sudden onset, without vomit, diarrhea or fever.

When he arrived to the ED, he was alert and complaining severe pain. His vital signs were: Blood Pressure 175/85, Heart Rate 70 rhythmic, Respiratory Rate 20/min, Oxygen Saturation 95% when breathing 28% Oxygen delivered by a Venturi Mask, temperature 35.2°. His skin was diaphoretic and mottled, without any peripherical edemas. The heart and lungs examination was normal. The abdomen was nondistended but hard and tender when palpating the epigastric and upper right quadrant. Bowel sounds were abolished.

While administrating morphine, an electrocardiogram, chest and abdominal X-ray, venous blood gases and complete blood test were taken, which results did not show any sign of acute illness. An abdominal tomography ruled out a bowel perforation, revealing gallstones without signs of cholecystitis and a hypodense focal lesion in the VI hepatic segment, compatible with liver abscess. Two sets of blood cultures (bioMerieux, France) were taken and incubated in a BacT/Alert Virtuo (bioMerieux, France) device. The patient was put on empiric antibiotic treatment with ceftriaxone and metronidazole, but 6 h after his arrival the pain persisted and his vitals began to deteriorate. A second blood test revealed the following results: white blood cell count 8.29 × 103/µL (2,3% of immature neutrophils); hemoglobin, 14.5 mg/dL; platelet count, 56 × 104/µ; aspartate transaminase, 1091 U/L; alanine transaminase, 684 U/L; gamma-glutamyl transpeptidase, 220 U/L; alkaline phosphatase, 213 U/L; total bilirubin, 4.95 mg/dL; conjugated bilirubin 3.60 mg/dl; amylase 392 U/L lipase 721 U/L; INR 2,40. The patient quickly developed a refractory septic shock and 12 h after, despite resuscitation with fluids and noradrenaline, he died.

**Fig. 1:**
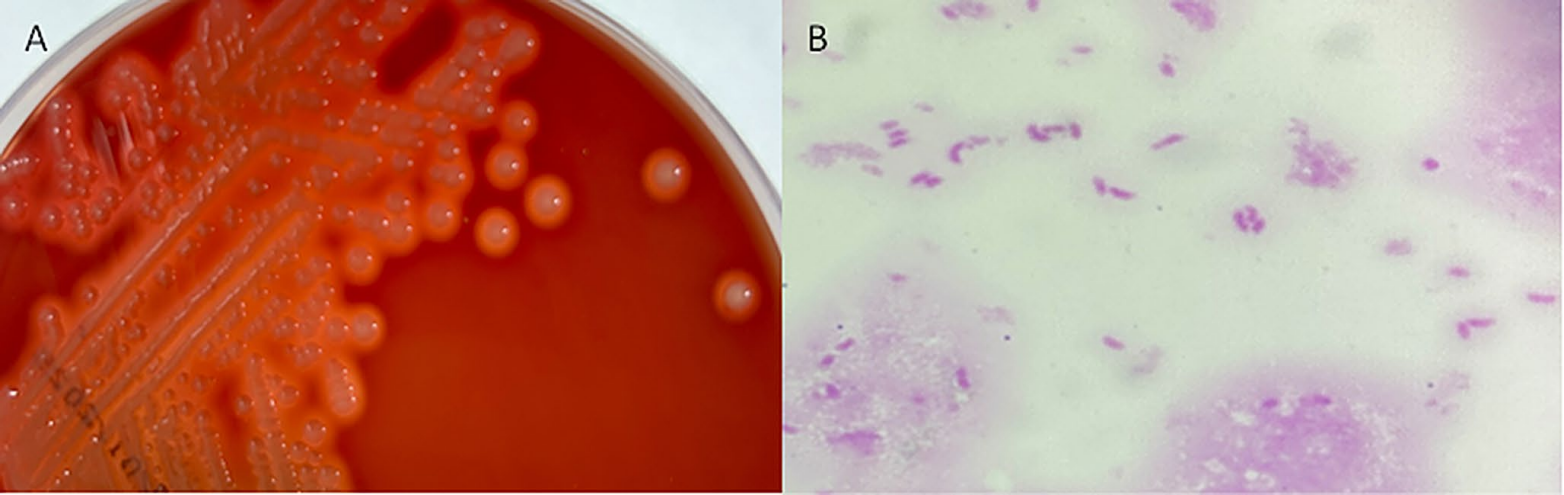
**A**) Vibrio fluvialis B-hemolytic colonies on 5% sheep blood agar after 24 h incubation. **B**) Gram stain of blood culture revealing curved Gram-negative bacilli

All blood culture bottles were positive for curved Gram-negative bacilli at 6 h of incubation and the isolate was identified by matrix-assisted laser desorption/ionization time-of-flight mass spectrometry (MALDI-TOF MS) using the MALDI using the MALDI Biotyper^®^ sirius GP System (Bruker Daltonik, Germany) as *V. fluvialis* with a score of 2.31 (Fig. 1). The identification was confirmed by whole-genome sequencing performed on an Illumina MiSeq system with 2 × 150 output. Raw reads were quality trimmed using fastp (available at https://github.com/OpenGene/fastp) and then assembled into contigs using shovill, with the SKESA methodology (available at https://github.com/tseemann/shovill and https://github.com/ncbi/SKESA, respectively). Assembled genome then was submitted to PubMLST Identification database (available at https://pubmlst.org/bigsdb?db=pubmlst_rmlst_seqdef_kiosk). The isolate exhibited β-hemolytic colonies when cultured on 5% sheep blood agar (bioMérieux, France) (Fig. 1).

Antimicrobial susceptibility testing was performed by the microdilution method using the commercial panel Thermo Scientific™ Sensititre DKMGN (Thermo Fisher Diagnostics, The Netherlands) or by gradient diffusion for azithromycin (Liofilchem, Italy) and doxycycline (bioMerieux, France) and interpreted according to the European Committee on Antimicrobial Susceptibility Testing (EUCAST) breakpoints. The isolate showed resistance to piperacillin-tazobactam and was susceptible to cefotaxime, ceftazidime, meropenem, ciprofloxacin, trimethoprim-sulfamethoxazole, azithromycin and doxycycline (Table 1).

**Table 1:** Antimicrobial susceptibility of the isolated *Vibrio fluvialis*

Antimicrobial	MIC (mg/L)	Interpretation
Piperacillin/tazobactam	4	R
Cefotaxime	≤ 0,5	S
Ceftazidime	≤ 0,5	S
Meropenem	≤ 0,12	S
Ciprofloxacin	≤ 0,06	S
Trimethoprim/sulfamethoxazole	≤ 1	S
Azithromycin	4	S
Doxycycline	0,5	S

MIC, minimal inhibitory concentration; S, susceptible; R, resistant

## Discussion

*V. fluvialis* infection’s typical clinical presentation is gastroenteritis. Nevertheless, an increasing incidence of extraintestinal infections has been reported, most commonly observed in insular or peninsular regions of Asia. These include otitis [11], wound infections [9, 10], necrotizing fasciitis [12], hemorrhagic cellulitis and cerebritis [13], cholangitis [14–17], peritonitis [18, 19], liver abscess [20], walled-off pancreatic necrosis [21], and urinary tract infections [22], in some cases with associated bacteremia [13–15, 20, 23–27]. A review of published literature on patients with *V. fluvialis* bacteremia highlighting key clinical and epidemiological features, antimicrobial treatment and outcome is summarized in Table 2.

**Table 2:** Summary of published cases of *Vibrio fluvialis* bacteremia

Country	Age (years), Sex	Diagnosis	Underlying condition	Exposure history	Treatment	Outcome	Author, year
Bangladesh	5 months, M	Gastroenteritis	Malnutrition	Not determined	Amoxicillin + Metronidazole Amoxicillin + Gentamicin + Pivmecillinam	Died	Albert, 1991
Taiwan	45, M	Hemorrhagic cellulitis cerebritis	Alcoholism	Wading in brackish water after fire-ant stings .	Oxacillin + Gentamicin Ceftazidime + oxytetracycline Fasciotomy + amputation	Died	Huang, 2005
Taiwan	65, M	Gastroenteritis	Diabetes mellitus, chronic liver disease	None	Cefuroxime Trimethoprim-sulfamethoxazole	Survived	Lai, 2006
USA	40, F	Gastroenteritis Catheter bacteremia	Diabetes mellitus, hypertension, end-stage renal disease on hemodialysis	Swimming in seawater	Ceftriaxone + Vancomycin + Doxycicline Gatifloxacin + Doxycicline Doxycicline	Survived	Nadkarni, 2007
Korea	70, M	Gastroenteritis	Gastric cancer, diabetes mellitus, hypertension	Not determined	Cefoperazone/sulbactam + Isepamicin	Survived	Koh, 2007
Korea	66, M	Not determined	Gastric cancer	Not determined	Not determined	Died	Koh, 2007
Japan	65, M	Liver abscess	Pancreatic head cancer	Seafood consumption (sashimi)	Piperacillin/tazobactam Piperacillin/tazobactam + Minocycline Abscess drainage, biliary stent	Survived	Kitaura, 2020
USA	64, M	Hemorrhagic skin bullae, gastroenteritis	Pancytopenia	Seafood consumption (raw oysters)	Piperacillin/tazobactam + Vancomycin Piperacillin/tazobactam + Daptomycin Piperacillin/tazobactam + Doxycycline Ceftriaxone + Doxycycline Ceftriaxone + Levofloxacin Doxycycline	Survived	Smith, 2023
Japan	72, M	Cholangitis	Hypertension, Hyperuricemia	Raw fish consumption (sushi chef)	Cefoperazone/sulbactam	Survived	Takezawa, 2024
Japan	78, F	Cholangitis	Diabetes mellitus, gallbladder-duodenal fistula	Sushi consumption	Ceftriaxone	Survived	Itoh, 2024
Spain	85, M	Cholangitis Liver abscess	Diabetes mellitus chronic obstructive pulmonary disease, dementia	None	Ceftriaxone + Metronidazole	Died	This case

Water or seafood exposure was noted in most of cases [5, 6]. Most of the patients affected by *V. fluvialis* bacteremia were middle-aged males [13, 15, 20, 24, 26, 27], many of them were affected by more than one comorbidity [14, 15, 24–26], primarily diabetes mellitus [14, 24–26], and approximately 35% of them died [13, 23, 26].

In our knowledge, *V. fluvialis* bacteremia has never been reported in Europe, while only one case of biliary infection with liver abscess has been reported worldwide, in Japan [20]. Our patient was a frail immunocompromised man with diabetes mellitus; the family denied salt water and seafood exposure, especially raw. The clinical presentation was atypical for cholangitis: he complained of severe epigastric and right upper quadrant pain but the first blood test was totally normal and he did not develop fever in any moment. In line with the high mortality of the documented extra intestinal infections, the prognosis of our patient was poor, quickly developing refractory septic shock and death despite optimal treatment. In our case, the patient was treated with ceftriaxone, to which the bacterium demonstrated susceptibility according to EUCAST breakpoint criteria. In previously documented cases of extraintestinal *V. fluvialis* infections, antimicrobial treatment strategies have been diverse, with both monotherapy and combination therapy commonly incorporating beta-lactams alongside aminoglycosides, quinolones, or tetracyclines, among the most frequently employe approaches [9, 11–16, 18–26].

The incidence of human *Vibrio* spp. infections is increasing worldwide [28]. Contributing factors to this trend include the global increase in seafood consumption, the internationalization of seafood trade, the expanding use of coastal waters for recreational purposes, and the effects of climate change, particularly rising sea surface temperatures, which promote the proliferation of *Vibrio* spp [28, 29]. As raw seafood consumption is getting more common than before, physicians should consider *V. fluvialis* infection alongside other *Vibrio* species in patients with exposure to seafood, especially if they are immunocompromised and/or affected by diabetes mellitus.

## Conclusions

Poor sanitation is the main risk factor for *Vibrio* species infections, but raw seafood consumption, which is increasing worldwide, represents another important one. Even though *Vibrio* infection usually causes acute gastroenteritis, extra intestinal infections have been reported and they should be taken into account, especially when dealing with diabetic patients. We described the first reported case in Europe of bacteremia due to *V. fluvialis*, causing the death of a frail immunocompromised patient for a hyperacute cholangitis complicated with liver abscess and refractory septic shock.

## Data Availability

No datasets were generated or analysed during the current study.
